# CO_2_ Is Beneficial to Gut Microbiota Homeostasis during Colonoscopy: Randomized Controlled Trial

**DOI:** 10.3390/jcm11185281

**Published:** 2022-09-07

**Authors:** Xue Yang, Wen-Bo Xiu, Jin-Xia Wang, Liang-Ping Li, Chong He, Cai-Ping Gao

**Affiliations:** 1 Department of Gastroenterology, Sichuan Academy of Science & Sichuan Provincial People’s Hospital, University of Electronic Science and Technology, Chengdu 610072, China; 2 Clinical Immunology Translational Medicine Key Laboratory of Sichuan Province, Sichuan Provincial People’s Hospital, University of Electronic Science and Technology, Chengdu 610072, China

**Keywords:** carbon dioxide, gut microbiota, probiotics, dysbiosis-related disease, colonoscopy

## Abstract

Background: Many studies have reported minor complications and disturbance of the gut microbiota after colonoscopy. Compared with air, carbon dioxide (CO_2_) insufflation could decrease minor complications, but its impact on gut microbiota remains unknown. Methods: Thirty-eight healthy subjects were assessed and twenty were randomized to receive either CO_2_ or air insufflation during colonoscopy. Neither the participants nor the staff involved in the follow-up knew which gas was used. Minor complications were assessed using symptom scores. Fecal samples were collected at eight time-points for microbiome analysis by full-length 16S rRNA gene amplicon analysis. Results: Baseline characteristics were similar in both groups. The recovery of minor complications after colonoscopy was faster in the CO_2_ group (the day of the colonoscopy) than in the air group (the day after the colonoscopy). There was no significant reduction in alpha diversity (species richness) of the first stool after colonoscopy in the CO_2_ group (115.0 ± 32.81 vs. 97.4 ± 42.31, *p* = 0.28) compared with the air group (123.8 ± 37.25 vs. 84.8 ± 31.67, *p* = 0.04). However, there were no differences in beta diversity between the groups. Linear discriminant analysis effect size (LEfSe) analysis indicated that anaerobic probiotics such as *Bacteroides caccae*, *Bacteroides finegoldii* and *Bacteroides thetaiotaomicron* were more abundant in the CO_2_ group than in the air group within 14 days after colonoscopy. On the contrary, the content of *Escherichia* *coli*, *Ruminococcus torques* and *Ruminococcus guavus* was higher in the air group. Conclusions: CO_2_ is beneficial to gut microbiota homeostasis during colonoscopy in healthy subjects. The effects in patients with different diseases need to be further studied.

## 1. Introduction

Over the last 30 years, colonoscopy has been widely used to improve the diagnostic and therapeutic utilities of the lower gastrointestinal system. In addition to suspected inflammatory bowel disease (IBD), hereditary polyposis syndrome, anemia, rectal bleeding, and unexplained chronic diarrhea, colonoscopy is also widely used for health checkups [1]. Complications of colonoscopy include serious complications, such as perforation and hemorrhage, and minor complications, such as abdominal pain, abdominal distension and diarrhea et al. [2]. Serious complications are rare now thanks to the continuous improvement of endoscopic equipment and the progress of endoscopic technology. However, minor complications occur frequently after colonoscopy. Additionally, may have an impact on a subject’s willingness to undergo colonoscopy again, especially in healthy subjects [3].

Colonoscopy also induces disturbance of the gut microbiota, also known as dysbiosis. Gut microbial dysbiosis can lead to the onset of many conditions ranging from gastrointestinal and metabolic diseases to neuropsychiatric and immunological diseases. Different results have been reported to demonstrate whether and/or when the dysbiosis returns to its pre- colonoscopy condition [4,5,6,7,8,9,10]. For example, Drago et al. have reported that colonoscopy prepared with a high-volume polyethylene glycol (PEG) had a long-term effect on gut microbiota composition and homeostasis in healthy subjects, particularly with a decrease in Lactobacillaceae abundance [9]. On the contrary, Jalanka et al. have reported that the dysbiosis could recover after colonoscopy, generally returning to baseline levels in 28 days and 14 days in young adults [8]. In addition, probiotic supplementation after colonoscopy may alter the gut microbiota, and alleviate minor complications [11,12].

Bowel preparation before colonoscopy and colon distension during colonoscopy are required. PEG is the most widely used intestinal cleanser [13]. The rapid and noticeable effects of PEG on minor complications and the dysbiosis have been demonstrated [8,9,10]. Bowel preparation is inevitable, so is there any way to reduce the disturbance of gut microbiota after colonoscopy?

The gut is an anaerobic environment. Currently, colonoscopy is performed mainly through air or carbon dioxide (CO_2_) insufflation. Air may destroy the anaerobic environment and aggravate the dysbiosis caused by bowel preparation. On the contrary, CO_2_ is more conducive to maintaining the anaerobic environment in the gut. CO_2_ insufflation has previously been reported to have advantages over air insufflation in terms of the minor complications after colonoscopy [12,13,14,15,16,17,18]. We hypothesized that CO_2_ may cause less disturbance to the gut microbiota than air during colonoscopy. In this randomized controlled trial (RCT), this effect was evaluated by comparing the gut microbiota of healthy subjects undergoing colonoscopy with CO_2_ or air insufflation.

## 2. Materials and Methods

**Study design:** This RCT was conducted at the department of Gastroenterology, Sichuan Academy of Science & Sichuan Provincial People’s Hospital, a tertiary referral center. Participants were recruited between August 2020 and April 2021. None of the participants were infected with COVID-19. This trial has been registered in the Chinese Clinical Trial Registry (Trial registration number: ChiCTR2000035218, http://www.chictr.org.cn/, accessed on 8 August 2020) and approved by the Ethics Committee of Sichuan Academy of Science & Sichuan Provincial People’s Hospital (NO. 2020404). Written informed consent for participation was obtained from all participants who were well informed about the study and potential risk.

**Figure 1:**
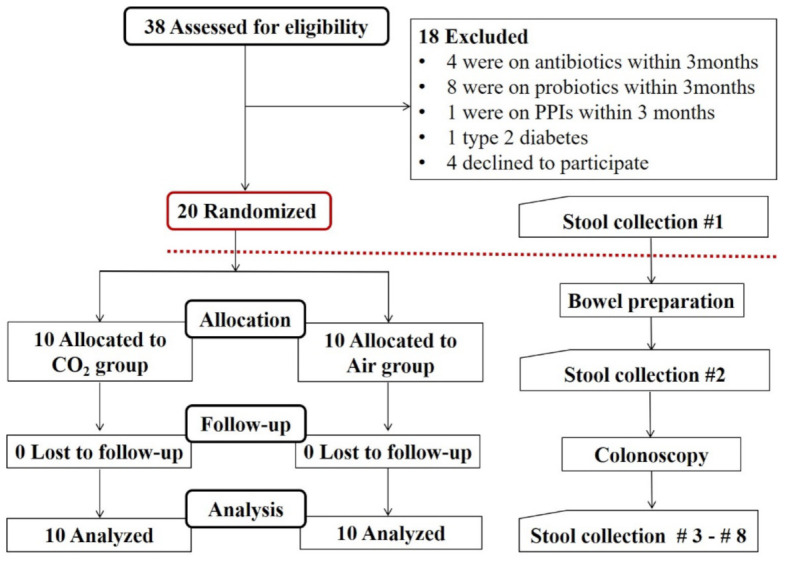
Flow chart of recruitment and randomization of healthy subjects undergoing colonoscopy with carbon dioxide (CO_2_) or air insufflation. PPI, Proton pump inhibitor. #3–#8, Stool samples were collected on the day of the colonoscopy and 1, 3, 7, 14, and 28 days after colonoscopy.

**Participants:** Thirty-eight healthy subjects were assessed (Figure 1). Inclusion criteria: (1) 18–60 years old, (2) no antibiotics used within 3 months, (3) no acid-suppressing drugs used within 3 months, (4) no yogurt, probiotics, prebiotics and health care products used within 3 months, (5) no drugs affecting intestinal motility used within 3 weeks, (6) informed consent was obtained, and (7) healthy participants with no GI symptoms. Exclusion criteria: (1) gastrointestinal surgery history, (2) malignant tumors, diabetes, thyroid dysfunction, liver and kidney dysfunction, (3) pregnancy, (4) intestinal disease, (5) PEG cleaning of the intestine is not acceptable, (6) colonoscopy reveals diseases that require the use of drugs and treatments that may affect the gut microbiota, such as tumors, IBD, and so on, and (7) serious adverse events occur during colonoscopy, such as perforation, bleeding, cardiac arrest, etc. Antibiotics, probiotics and acid-blocking drugs were banned, and participants were asked to avoid alcohol, high-fat and spicy foods during the trial.

**Randomization and the endoscopic procedure:** Twenty healthy subjects were enrolled and randomly allocated to receive either CO_2_ (CO_2_ group, 10 subjects) or air (air group, 10 subjects) insufflation. The randomization code was computer generated. Participants were randomized in sequence according to the randomization code and arranged to have colonoscopy according to a randomization table. All participants were asked to ingest two liters of PEG solution 4–6 h before colonoscopy. An expert endoscopist (>10 years of experience) and endoscopy assistants were not blinded to the type of gas used. Staff who were blinded to the trial assignment were involved in follow-up visits. All participants were also blinded to the type of gas used and sedated with intravenous propofol and midazolam. The target sedation level was general anesthesia. Morning colonoscopies were performed using a CF-Q290AL colonoscope (Olympus Medical Systems Corp, Tokyo, Japan). CO_2_ was administered using a commercial CO_2_ endoscopic insufflator (Olympus Medical Systems Corp) connected to a CO_2_ tank.

**Minor complications:** According to previous studies, participants may suffer minor complications, such as abdominal pain, abdominal distension, diarrhea, constipation, defecation discomfort and mucus stool after colonoscopy [14,15,16]. In the current study, each symptom was divided into four grades: none, mild, moderate and severe, and the changes of symptoms before and after colonoscopy were evaluated according to 0, 1, 2 and 3 scores, respectively. The total score was 0~18 points (Table 1). Symptom assessment time was consistent with fecal sample collection.

**Fecal sample collection:** Participants were asked to collect fecal samples in provided sterile containers before bowel preparation, after bowel preparation, the day of the colonoscopy, and the 1st, 3rd, 7th, 14th, and 28th days after colonoscopy (Figure 1). Stool samples collected at home were immediately delivered to the hospital within 1 h for storage at −80 °C. Samples collected on the day of colonoscopy were immediately frozen at −80 °C.

**Full-length 16S rRNA sequence analysis:** The changes in gut microbiota were analyzed using full-length 16S rRNA gene amplicon analysis. The PowerSoil^®^ DNA Isolation kit was used for bacterial genomic DNA extraction following the manufacturer’s instructions. The 16S full-length gene was amplified with PCR by using specific primers 27F (AGRGTTTGATYNTGGCTCAG) and 1492R (TASGGHTACCTTGTTASGACTT). Then, the quality of the sequencing library was tested, and the circular consensus sequencing (CCS) sequence was barcoded. The generated optimization CCS was clustered at a 97% similarity level (USEARCH, Version 10.0) and its species classification was classified based on the sequence composition of operational taxonomic unit (OTU). Species annotation, classification and diversity analysis of gut microbiota was performed using the 16S: Silva database and RDP classifier. Species abundance and the diversity of individual samples were analyzed using Alpha diversity analysis. The differences in community composition and structure among different samples were compared by Beta diversity analysis. Linear discriminant analysis effect size (LEfSe) was used to screen biomarkers with statistical difference between the CO_2_ and air group (biomarker screening criteria: LDA score > 4).

**Statistical analysis:** Due to the large difference in gut microbiota between individuals, there is no normal value for sample size calculation. The sample size of this study was referenced by other similar studies [17,18]. Data were analyzed with SPSS version 22.0. Chi-square test or Fisher’s exact test were used for counting data. Continuous data were expressed as X ± SD, and comparisons between groups were analyzed by Student’s *t* test and Pairwise comparison analysis of variance. LEfSe analysis was performed to search for differential biomarkers. *p* < 0.05 was statistically significant.

## 3. Results

**Characteristics of the participants:** A total of 20 healthy subjects participated in this study. Among them, 10 participants were allocated into the CO_2_ group, and 10 participants were allocated into the air group. All of them completed the study (Figure 1).

Colonoscopy was performed and fecal samples from all participants were collected for comparative analyses (Table 2). There were no differences between the two groups in age, male/female, body mass index (BMI), total procedure time (insertion and withdrawal), positive results (polyps), or number of fecal samples. The Boston scores of all subjects during colonoscopy were 6 or above, except for one subject. For one subject of the CO_2_ group, colonoscopy lasted for about 10 min and failed to reach ileocecum due to unsatisfactory bowel preparation. An analysis found that regardless of whether this case was excluded or not, the results remained the same.

**Figure 2:**
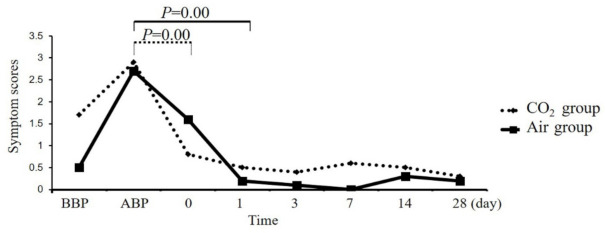
Changes in minor complications. Although there was no difference in minor complications between the CO_2_ and air groups at each follow-up point (Table 2), the recovery of minor complications after colonoscopy was faster in the CO_2_ group (2.9 ± 0.99 vs. 0.8 ± 1.48, *p* < 0.01) than in the air group (2.7 ± 0.68 vs. 1.6 ± 1.90, *p* = 0.10). CO_2_, carbon dioxide. BBP, before bowel preparation. ABP, after bowel preparation. 0, 1, 3, 7, 14, 28 indicates the day of the colonoscopy and 1, 3, 7, 14, 28 days after colonoscopy.

**Minor complications:** There was no difference in symptom assessment between the CO_2_ group and the air group before and after bowel preparation (Figure 2). Symptom scores increased in both groups after bowel preparation. Notably, symptom scores of the CO_2_ group began to decrease on the day of the colonoscopy, which were different from those after bowel preparation (2.9 ± 0.99 vs. 0.8 ± 1.48, *p* < 0.01. Figure 2). However, symptom scores of the air group did not decrease significantly on the day of the colonoscopy, which were not different from those after bowel preparation (2.7 ± 0.68 vs. 1.6 ± 1.90, *p* = 0.10. Figure 2), and then gradually recovered on the day after colonoscopy (2.7 ± 0.68 vs. 0.2 ± 0.42, *p* < 0.01. Figure 2). Symptom scores showed no significant difference between the two groups from the first day to the 28th day after colonoscopy (Figure 2). The results suggested that minor complications of the CO_2_ group recovered faster after colonoscopy than that of the air group.

**Changes in the gut microbiota:** After 144 samples were sequenced, a total of 950,441 CCS sequences were obtained through barcode identification. Each sample produced at least 4564 CCS sequences, with an average of 6000 CCS sequences.

**Figure 3:**
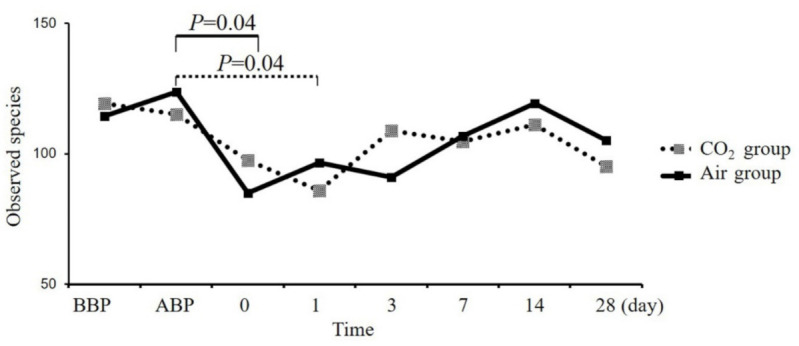
Changes of the species richness (alpha diversity) of gut microbiota in the CO_2_ group and the air group. The species richness after colonoscopy decreased more slowly and recovered more quickly in the CO_2_ group (115.0 ± 32.81 vs. 97.4 ± 42.31, *p* = 0.28) than in the air group (123.8 ± 37.25 vs. 84.8 ± 31.67, *p* = 0.04). CO_2_, carbon dioxide. BBP, before bowel preparation. ABP, after bowel preparation. 0, 1, 3, 7, 14, 28 indicates the day of the colonoscopy and 1, 3, 7, 14, 28 days after colonoscopy.

At baseline, there were no significant differences in alpha diversity between the CO_2_ group and the air group (Figure 3). The alpha diversity (species richness) decreased after colonoscopy in both groups. Notably, in the CO_2_ group, there was no significant decrease in alpha diversity on the day of the colonoscopy (115.0 ± 32.81 vs. 97.4 ± 42.31, *p* = 0.28. Figure 3), and it decreased gradually on the day after colonoscopy (115.0 ± 32.81 vs. 85.8 ± 34.16, *p* = 0.04. Figure 3). However, the alpha diversity of the air group decreased significantly on the day of the colonoscopy (123.8 ± 37.25 vs. 84.8 ± 31.67, *p* = 0.04. Figure 3). Although there was a trend for restoration of microbiota with time in both groups, the alpha diversity of the CO_2_ group was recovered faster than that of the air group (Figure 3). Compared with baseline, the species richness (alpha diversity) was reduced after colonoscopy, but the abundance returned to baseline at 14 and 28 days after colonoscopy in both groups (Figure 3). These results suggest that CO_2_ insufflation interferes less in the richness of the gut microbiota than air during colonoscopy.

**Figure 4:**
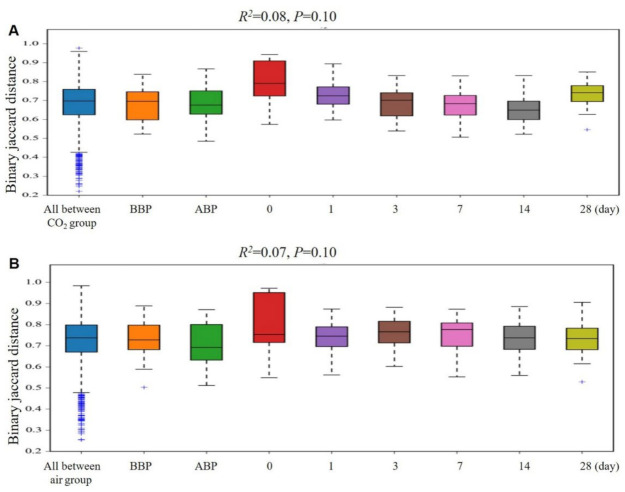
Changes of the gut microbiota composition (beta diversity) in the CO_2_ group and the air group. No differences in the gut microbiota composition after colonoscopy within the CO_2_ group (**A**) and the air group (**B**) or between two groups at each time point (data not shown). CO_2_, carbon dioxide. BBP, before bowel preparation. ABP, after bowel preparation. 0, 1, 3, 7, 14, 28 indicates the day after colonoscopy and 1, 3, 7, 14, 28 days after colonoscopy.

There were no significant differences in the beta diversity between the CO_2_ group and the air group. Additionally, compared with baseline, there were no significant differences in the gut microbiota composition (beta diversity) within 28 days after colonoscopy in both groups (Figure 4).

**Figure 5:**
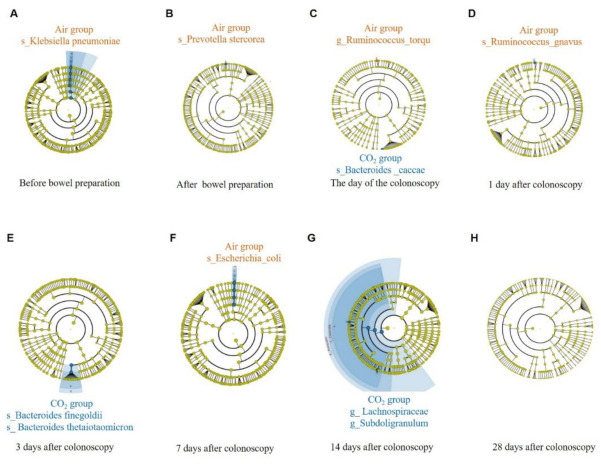
Differences in intestinal bacteria after colonoscopy. Cladogram of LEfSe analysis showed that within 14 days after colonoscopy, *Bacteroides caccae*, *Bacteroides finegoldii*, *Bacteroides thetaiotaomicron*, Subdoligranulum and *Lachnospiraceae* in the CO_2_ group were significantly higher; On the contrary, *Escherichia coli*, *Ruminococcus torques* and *Ruminococcus guavus* in the air group were significantly higher. CO_2_, carbon dioxide.

In order to further study the effects of CO_2_ and air on the gut microbiota, LEfSe analysis was performed to search for differential biomarkers, and identified eight significantly different species and two different genera between the two groups. Within 14 days after colonoscopy, *Bacteroides caccae*, *Bacteroides finegoldii*, *Bacteroides thetaiotaomicron*, Subdoligranulum and *Lachnospiraceae*, which were all obligate anaerobes, in the CO_2_ group, were significantly higher compared with that in the air group. On the other hand, *Escherichia coli*, *Ruminococcus torques* and *Ruminococcus guavus* in the air group were significantly higher than that in the CO_2_ group (Figure 5). Although there were more *Prevotella Stercorea* and *Klebsiella pneumoniae* in the air group than in the CO_2_ group before colonoscopy, there was no difference between the two groups after colonoscopy (Figure 5). These results suggested that CO_2_ was more conducive to the growth of obligate anaerobes during colonoscopy than air.

## 4. Discussion

To our knowledge, this is the first RCT comparing CO_2_ with air insufflation on gut microbiota during colonoscopy, demonstrating that: (1) minor complications after colonoscopy recovered more quickly in the CO_2_ group; (2) Smaller changes and faster recovery of the alpha diversity of the gut microbiota after colonoscopy with CO_2_; (3) Colonoscopy with CO_2_ is beneficial to the growth of anaerobic probiotics, such as *Bacteroides caccae*, *Bacteroides finegoldii*, *Bacteroides thetaiotaomicron* et al., and a decrease in *Escherichia coli*, *Ruminococcus* *torques* and *Ruminococcus guavus* after colonoscopy in the CO_2_ group compared with the air group. These observations suggest that CO_2_ may be a better choice for colonoscopy in healthy subjects.

Gut microbiota homeostasis is an important factor in maintaining intestinal mucosal barrier and immune function. Whether short-term disturbance in gut microbiota increases disease risks or not is not clear. However, it has been found that people concurrently exposed to antibiotics and bowel preparations were more likely to suffer irritable bowel syndrome after colonoscopy [19]. Therefore, reducing the degree of gut microbiota disturbance and shortening the duration of the disturbance is beneficial to reducing potential risks.

A number of studies have observed the effects of bowel preparation and colonoscopy on gut microbiota. Among them, most found the disturbance of gut microbiota after colonoscopy [4,5,7,8], except O’Brien et al., who reported that bowel preparation did not have an effect on the composition of the gut microbiota for the majority of subjects [6]. Furthermore, that disturbance of gut microbiota was related to minor complications after colonoscopy [7]. About one third of people suffer minor complications after colonoscopy [3], and that could even lead to sick leave in a minor subgroup. Therefore, it is necessary to optimize colonoscopy to minimize minor complications and gut microbiota disturbances.

This study found CO_2_ insufflation increased the relative abundance of some Bacteroides members, such as *Bacteroides caccae*, Subdoligranulum and *Lachnospiraceae*, which are all anaerobic and probiotics (Figure 5). *Bacteroides caccae* was correlated with the levels of IgA in the gut, which might protect against bowel pathogens [20]. *Bacteroides caccae* was also correlated with immune checkpoint inhibitor activity in metastatic melanoma patients [21]. Subdoligranulum has been found to ferment in the gut to produce short-chain fatty acids, which were generally considered to have a variety of important effects on maintaining health [22]. Lachnospiraceae might contribute substantially to the radioprotection of the hematopoietic system and intestinal system [23]. On the contrary, air insufflation increased the relative abundance of *Escherichia coli*, *Ruminococcus* *torques* and *Ruminococcus guavus*. *Escherichia coli* is facultative anaerobic bacteria, which grows well under either oxic or anoxic conditions. Studies have reported that the abundance of *Ruminococcus torques* in the gut of patients with autoimmune diseases, such as IBD, Hashimoto's thyroiditis and amyotrophic lateral sclerosis, was significantly increased [24,25,26], whereas the abundance of Bacteroides was decreased in Hashimoto's thyroiditis patients [23]. Studies have also reported the abundance of *Ruminococcus guavus* enriched in the gut of patients with IBD [27], and intestinal dysbiosis with increased *Ruminococcus gnavus* abundance associated with allergic diseases in infants [28]. Therefore, we can speculate that CO_2_ insufflation is beneficial to the growth of anaerobic probiotics, reducing the disturbance of gut microbiota after colonoscopy, and may reduce potential risks caused by the disturbance of gut microbiota.

Our study has two limitations. One limitation is that, as with other similar studies [17,18], there were only 10 participants in each group. Although stool samples from each participant were analyzed at multiple time points before and after colonoscopy, the clinical significance needs to be further studied with expanded samples in the future. There is an ongoing multicenter study to confirm these preliminary results. Another was that our study only observed healthy adults, while people from different regions or with different diseases have different gut microbiota, which may lead to different responses to CO_2_ or air insufflation during colonoscopy. Therefore, the beneficial effect of CO_2_ on gut microbiota needs to be further verified by expanding the sample size of different populations.

## 5. Conclusions

Our results suggest that, in addition to reducing minor complications, CO_2_ might also be beneficial to the gut microbiota homeostasis during colonoscopy in healthy subjects. The effects on patients with different diseases need to be further studied.

## Figures and Tables

**Table 1:** Minor complications were assessed as following symptom scores.

Symptoms	Grade	Points
Abdominal pain	None	0
	Mild	1
	Moderate	2
	Severe	3
Abdominal distension	None	0
	Mild	1
	Moderate	2
	Severe	3
Diarrhea	A normal number of daily stools	0
	One to two more stools than normal	1
	Three to four more stools than normal	2
	Five or more stools than usual	3
Constipation	A normal number of daily stools without difficult defecation	0
	Mild	1
	Moderate	2
	Severe	3
defecation discomfort	None	0
	Mild	1
	Moderate	2
	Severe	3
mucus stool	None	0
	Mild	1
	Moderate	2
	Severe	3

**Table 2:** Participant and examination characteristics.

		CO_2_ (n = 10)	Air (n = 10)	*p* Value
**Age, mean ± SD, years**		42.6 ± 7.2	40.4 ± 8.2	0.53
**Male/Female**		2/8	3/7	1.00
**BMI, mean** **±** **SD, kg/m^2^**		22.9 ± 2.3	22.3 ± 2.0	0.52
**Procedure time, mean ± SD, minutes**				
Total procedure time		10.7 ± 1.3	10.5 ± 1.3	0.73
Time to cecal intubation		4.4 ± 1.3	4.0± 1.1	0.45
**Completion of colonoscopy**				
	Finished	10	10	1.00
**Results**				
	Negative	6	6	1.00
	Positive	4 Polyps	3 Polyps	1.00
**Complications**	BBP	1.7 ± 2.45	0.5 ± 0.71	0.17
	ABP	2.9 ± 0.99	2.7 ± 0.68	0.61
	0 d	0.8 ± 1.48	1.6 ± 1.90	0.31
	1 d	0.5 ± 0.71	0.2 ± 0.42	0.27
	3 d	0.4 ± 0.84	0.1 ± 0.32	0.31
	7 d	0.6 ± 1.35	0.0 ± 0.00	0.19
	14 d	0.5 ± 1.27	0.3 ± 0.68	0.67
	28 d	0.3 ± 0.68	0.2 ± 0.63	0.74
**Fecal samples**		7.1 ± 0.7	7.3 ± 0.9	0.61

BMI, body mass index. BBP, before bowel preparation. ABP, after bowel preparation. 0d, 1d, 3d, 7d, 14d, 28d indicates the day after colonoscopy and 1, 3, 7, 14, 28 days after colonoscopy.
